# The Role of Pelvic Reirradiation in the Treatment of Locally Recurrent Rectal Cancer: A Systematic Review

**DOI:** 10.3390/biomedicines14061194

**Published:** 2026-05-25

**Authors:** Rachael E. Clifford, Sulaimaan Hannan, Hamish W. Clouston, Victoria Lavin, Claire Arthur, Paul A. Sutton

**Affiliations:** 1 Colorectal and Peritoneal Oncology Centre (CPOC), The Christie NHS Foundation Trust, Manchester M20 4BX, UK; 2 Division of Cancer Sciences, Faculty of Biology, Medicine and Health, The University of Manchester, Manchester M13 9PT, UK

**Keywords:** rectal cancer, recurrent rectal cancer, radiotherapy, reirradiation

## Abstract

**Background:** Local recurrence of rectal cancer is a challenging problem for patients and clinicians. Surgical resection is associated with good outcomes if R0 margins are achieved; however, it is often complex, requires suitable patient fitness, and is associated with long term physical and psychological consequences. Meanwhile, continuing technical advances in radiotherapy have enabled the delivery of highly conformal treatment, thereby enabling dose escalation or pelvic reirradiation to be safely considered—either as definitive management or in the neoadjuvant setting—for patients with locally recurrent rectal cancer. Pelvic reirradiation may refer to patients who have received primary rectal radiotherapy with the aim of neoadjuvant downstaging or reducing the risk of locoregional recurrence, versus radiotherapy for a previous unrelated non-rectal pelvic malignancy. **Methods:** A literature search of pelvic reirradiation for non-metastatic, locally recurrent rectal cancer was conducted for full text articles published over the last 20 years. Additional papers were identified within the references of these papers. Studies focusing on non-rectal cancers, and patients having primary radiotherapy for locally recurrent rectal cancer were excluded. Due to the heterogenicity of the data, no meta-analysis was performed. **Results:** A total of 15 papers were included, containing a cohort of 840 patients. Several reirradiation modalities were reported, including external beam radiotherapy, brachytherapy, stereotactic ablative radiotherapy and heavy particle therapy (carbon ion). Carbon ion radiotherapy was the most common reirradiation treatment modality utilised with a median cumulative dose of 70.4 Gray (Gy). Treatment response, defined as either complete or partial improvement in tumour size, was only reported in seven studies, and varied from 14 to 88%. Overall 3-year survival was also variable with rates reported between 18 and 85%. These observations may be due to variation in patient selection, treatment intent, and technique. Pelvic reirradiation was associated with acceptable toxicity, low rates of G3+ toxicity, and improved symptom control. **Conclusions**: Our review describes the multitude of approaches to pelvic reirradiation for locally recurrent rectal cancer. Reviewing the radiobiological and patient outcomes is challenging in view of the degree of heterogeneity in patient selection, treatment approach, and reported outcomes. However, there is consensus that pelvic reirradiation—either for long term control or to downstage prior to definitive surgery—is feasible with potential utility in this setting.

## 1. Introduction

There are over 44,000 new cases of colorectal cancer diagnosed in the UK each year, with approximately one third of these originating in the rectum [1]. Globally, rates have more than doubled since the 1990s [2] with an associated increase in incidence in younger age groups representing a ‘third peak’ [3]. The management of rectal cancer has improved significantly in the same time frame, with total mesorectal excision (TME), improvements in peri-operative staging, and advances in neoadjuvant and organ preservation strategies [4].

With respect to long term outcomes, up to 20% of cancers recur with either local or distant disease within five years of surgical intervention [5]. Locally recurrent rectal cancer (LRRC) occurs at any time following primary resection within the pelvis [6] and is often asymptomatic and identified on surveillance imaging [7]. Locally recurrent rectal cancer is associated with a 5-year overall survival (OS) of up to 31.1% [8] and can be anatomically classified using the Memorial Sloan–Kettering criteria, first described in 1984 and further developed 20 years later by Moore [9]. This classification is based on anatomical location within the intra-pelvic compartment, divided into axial, anterior, posterior, or lateral compartments, and is used to tailor treatment options. Where feasible and with a realistic prospect of achieving an R0 resection, surgery is usually the first line approach, although tumours in the axial compartment may be more amenable to this than in the other compartments [9].

In cases where clear margins cannot be achieved, or there is concern over patient fitness and long-term functional outcomes, alternative oncological strategies with either neoadjuvant or palliative intent can be considered. Nearly half of patients who present with LRRC will receive treatment with palliative intent, achieving a 5-year OS rate of 10–30% [10]. Arguably, however, the aim of treatment is to offer long term symptom control rather than improve survival.

Pelvic reirradiation has been a growing area of clinical interest [11]. Reirradiation techniques include external beam radiotherapy (EBRT), brachytherapy, stereotactic ablative radiotherapy (SABR), and heavy particle therapy including carbon ion radiotherapy (CIRT). The challenge with reirradiation starts with acknowledging the lack of consensus of an agreed definition of ‘reirradiation’—with regards to the degree of overlap with previous radiation fields, the time interval between treatments (which enables repair of normal tissue), and treatment modality. In 2022 the European Society for Radiotherapy and Oncology (ESRO) published a consensus document aiming to standardise some of these areas for future prospective studies and to allow cross study comparisons [12]. Potential reirradiation strategies will be guided by the intent of previous radiotherapy, modality, dosing, treatment volume, and time interval between treatments. Radiotherapy late effects and cumulative dose to organs at risk (OAR) are critical in determining a safe reirradiation dose.

This study aims to systematically review the current literature on the use of reirradiation in the management of LRRC in both curative and palliative settings to consolidate current understanding of indications, limitations, and outcomes.

## 2. Methods

This systematic review was registered with PROSPERO (ID: CRD42023403871). No meta-analysis was planned or performed due to expected heterogenicity of the studies included.

A literature search was formed using the search string (palliative) OR (re-irradiation) OR (reirradiation) OR (radiotherapy) OR (oncology) OR (recurrence) OR (SABR) OR (stereotactic ablative body radiotherapy) OR (EBRT) or (external beam radiotherapy) or (IMRT) or (Intensity Modulated Radiotherapy) or (Intensity Modulated Radiation Therapy) AND (recurrent rectal cancer) OR (recurrent rectal neoplasm) OR (recurrent rectal tumour) OR (palliative rectal cancer)’ in the PubMed/EMBASE and Cochrane Library in January 2024. An expanded search strategy is included in Supplementary Table S1. Studies were included that reported on LRRC and the use of any form of reirradiation therapy and were published within the last 20 years. Studies including non-rectal cancers and patients having first line surgery or primary radiotherapy for LRRC were excluded. Papers not available in the English language and those where full text was not available were also excluded, as were systematic reviews. Additional papers were identified by scanning the references of eligible papers. Risk of bias was assessed in each study using the Cochrane RoB-2 tool for randomised studies and ROBINS-I tool for non-randomised studies. The systematic review PRISMA checklist is included in Table S2.

## 3. Results

**Figure 1:**
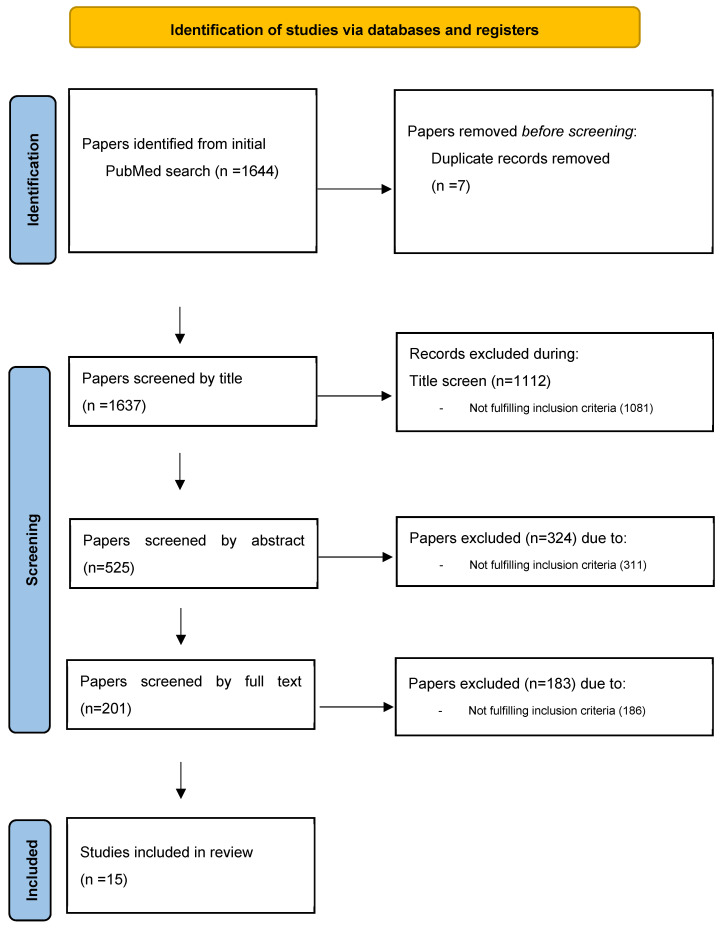
PRISMA flow diagram demonstrating study exclusion. Fifteen studies met the inclusion criteria for this systematic review.

Fifteen studies were included in this review, which has been reported in line with the Preferred Reporting Items for Systematic Reviews and Meta-Analyses (PRISMA) (Supplementary Table S2). The PRISMA flow diagram is included as Figure 1. All included studies were deemed to have high potential risk of bias. A total of 840 patients were reported within the eligible studies, ranging from 7 to 173 in each included study. A number of reirradiation techniques and approaches were described; a summary of the primary and reirradiation treatments, including dose, location of disease, and reirradiation volume can be seen in Table 1. Cumulative radiotherapy dose and EQD2 calculations (if appropriate) are also shown. The EQD2 is a simplified dose calculation using the linear quadratic model which enables comparisons between different dose fractionation schedules and calculation of cumulative dose. EQD2 has some important limitations; primarily, it does not account for dose homogeneity, variability in field overlap, or recovery of normal tissues between retreatments.

### 3.1. Treatment Modality

Of the studies included, six utilised EBRT, three SABR, five CIRT and one interstitial brachytherapy. The EBRT cohort encompassed a range of approaches including intensity modulated radiotherapy (IMRT), 3D conformal, radiotherapy alone, or radiotherapy combined with concurrent chemotherapy as a radiosensitiser.

### 3.2. Survival

Across all studies, median follow up was 30 months, ranging from 22 to 65 months. The 3-year overall survival (OS) ranged from 18 to 86%, with the highest figure reported by Chung et al. with CIRT at a median total dose of 70.4 Gray (Gy) across 16 fractions [24]. Conversely, the lowest reported 3-year overall survival rate was 18% by Cai et al. with EBRT, who also had the greatest grade 3 toxicity rate [16]. Two studies reported 5-year OS ranging from 38 to 65%. Survival, local control, and toxicity data are summarized in Table 2.

### 3.3. Treatment Response

Treatment response, defined as either complete or partial improvement in tumour size, was only reported in seven studies, and varied from 14 to 88%. The highest rate of 88% was achieved in a cohort of 56 patients who received once daily fractionations of radiotherapy, most commonly 39.6 Gy in 22 fractions of 1.8 Gy, and a range of 20 Gy/10 to 39.6 Gy/22 [14]. It is worth noting, however, that 73% of these patients were treated with palliative intent.

### 3.4. Local Failure

Local failure, defined as progression of local disease on treatment, ranged from 11 to 84%. The lowest local failure rate of 11% was achieved in a cohort of 18 patients who received SABR at a median dose of 25 Gy/5# [20]. The highest local failure rate of 84% was seen in a cohort of 33 patients who received a median total dose of 30 Gy via both 2D and 3D conformal radiotherapy [13].

### 3.5. Distant Failure

Distant failure, defined as the progression or development of metastatic disease, was reported in 10 of the 15 included studies, ranging from 16 to 71%. The lowest rate of distant failure of 16% was seen in two studies, with one cohort receiving SABR 30 Gy/5# and the other receiving CIRT 36–51 Gy/12–18#. The highest rate of distant failure of 71% was seen in a cohort that received CIRT 70.4 Gy/16#.

### 3.6. Treatment Compliance and Toxicity

Data on patient compliance with treatment and toxicity were also reviewed where available. Patient compliance was reported in 14 of the 15 included studies, 12 of which reported 100% compliance. A further paper reported 91% compliance in patients receiving accelerated hyper-fractionated intensity modulated radiotherapy at a total dose of 39 Gy [25]. The final paper reported 86% compliance in those receiving preoperative hyper-fractionated chemoradiation at a total dose of 40.8 Gy [27].

## 4. Discussion

Locally recurrent rectal cancer is a complex and challenging problem. Although the technical feasibility of R0 resection is often the first consideration given its favourable outcomes, this surgery is morbid for a number of reasons, including previous oncological therapy, prior surgery, and the extended nature of the resections. Patient selection is key, and a multidisciplinary approach is needed for appropriate assessment of fitness, peri-operative counselling, and consent. Due to these challenges, alternative oncological strategies that are highly efficacious are desired, both in the neoadjuvant and palliative setting.

Selection of optimal treatment, both modality and dose, is guided by previous radiation target organ, prior modality, dose, treatment volume, time interval between treatments, and location of recurrence. Previous pelvic radiotherapy may not have been targeted at a primary rectal cancer but instead an alternative primary pelvic malignancy e.g., prostate or cervix. By virtue of radiotherapy volume expansions (allowing for microscopic disease and patient motion), treatment plan conformality, or radiation beam divergence, the rectum will have been included within the irradiated field. The location of pelvic recurrence will likely determine the reirradiation dose; for example, a higher dose may be delivered to lateral nodal disease or disease overlying muscle or bone when compared to luminal recurrence, as the likelihood of acute and/or late toxicity is lower [28]. Ultimately, the choice of modality is often determined by availability within healthcare systems. Whilst EBRT and IMRT are commonly available, SIRT is not.

SABR is a promising alternative treatment modality being increasingly utilised for oligometastatic disease. High-dose conformal radiation is delivered in three to five fractions, which in a selected cohort establishes good local control with minimal local toxicity [29]. It is particularly useful for low volume, non-luminal recurrence, such as nodal disease. Its role has been explored in local recurrence for patients not fit for surgery; however, it is yet to be established as a first line option where surgery is available [20]. Different patterns of recurrence, with respect to both location and size, will need different planning approaches.

Brachytherapy has an established role as a contact therapy in other pelvic malignancies, such as prostate and gynaecological. By virtue of its radiobiology traits, brachytherapy can deliver high doses directly to the tumour (e.g., rectal lumen) with rapid dose fall off ensuring adjacent normal tissues can be spared. Proton beam has an established role in paediatric and ocular malignancies, but as yet it has no first line role for rectal cancer outside of the trial setting.

This review has demonstrated the potential increasing role of reirradiation for locally recurrent rectal cancer in the first line setting, although the currently available literature precludes us from drawing any definitive guidance. As disease biology is better understood and radiotherapy techniques evolve, survival rates improve, with 3-year OS reaching as high as 86%. Furthermore, disease-free survival with local and distant control is also very encouraging. It is important to acknowledge that some of the variation observed between the studies may be due to differences in patient selection, treatment intent, and technique. Susko et al. [13] was the only paper to compare reirradiation modalities, finding no significant differences between treatment with EBRT (comparing 2D/3D conformal and IMRT), and intraoperative radiotherapy (IORT). This suggests that reirradiation for rectal cancer is possible with encouraging results, regardless of the radiation modality utilised. The authors acknowledge that further work is needed in order to determine whether the use of IMRT is more beneficial than 3D conformal radiotherapy with respect to treatment toxicity and response.

Used as a neoadjuvant adjunct, reirradiation would be downstaging the tumour in order to increase the chances of a R0 resection margin. Although this offers a potential option for patients who are fit with borderline resectability, it has been shown to be associated with a higher rate of late toxicity in those who subsequently undergo surgery [30]. Within the literature, it is often difficult to establish the initial intent of treatment. Susko et al. [13] is one of only a few studies which report the proportion of patients progressing to surgical resection; 42% of patients underwent resection with a 71% R0 rate. Valentini et al. reported a partial pathologic response rate of 36%, and a complete pathologic response (pCR) rate of 9% among patients who had surgery after re-irradiation [18]. The combination of reirradiation and surgery for the management of LRRC represents an opportunity for patients to benefit from both treatment modalities, although the impact of reirradiation on surgery is still not yet fully understood with respect to either peri-operative morbidity or pathological/oncological outcomes. As previously discussed, R0 resection remains the single biggest predictor of outcomes following surgery, and in their paper, Suko et al. have shown an R0 rate comparable to the PELVEX dataset of patients with LRRC proceeding directly to surgery [13,31]. Whilst clearly subject to bias in case selection, these data do suggest that optimal rates of R0 resection can be achieved in the post reirradiation pelvis. Reirradiation as primary treatment may allow patients to avoid the need for further surgical intervention in a challenging pelvic field. Surgical adhesions, fibrosis from neoadjuvant therapy, and the confined spaces within the pelvis all increase the potential morbidity of re-operation.

The aim of palliative reirradiation is two-fold; gain local control, which may result in improvements to overall survival (with or without subsequent surgery), and symptom control. For patients treated with palliative intent with a median dose of ≥30 Gy, the proportion of patients gaining partial or complete pain relief ranges from 83 to 94%, with more than 80% of patients experiencing relief from gastrointestinal symptoms for a median of nine months [32]. Acute toxicity has improved over time with increasingly conformal radiotherapy and smaller margins. Ng et al. reported the highest rate of radiological treatment response in over 88% of patients [14], 73% of which, however, were treated with palliative intent. Whilst this is an important outcome in terms of OS, it is their 91% completion rate and 88% 3-month symptom response rate which are the most impressive outcomes in this cohort; however, limited data on late toxicity are reported.

Warrier et al. reported that Quality of Life (QoL) measures stabilised following exenterative surgery compared to a gradual decline with palliative care alone [8,33]. Outcomes and survival rates are known to be significantly improved following R0 resection [8], with local recurrence rates of 4-10%. In studies published on reirradiation, QoL data is acutely lacking with limited reporting on toxicity in six papers. The included studies do, however, report an encouragingly high compliance rate, with the lowest reported at 81% by Ng et al. [14] detailing a 12.5% rate of grade 3 (serious adverse event requiring medical intervention or hospitalisation) acute toxicity for patients receiving CIRT at a total dose of 39.6 Gy, with one patient palliated for a late complication. The time interval between initial and re-treatment will likely play a key role in establishing the viability of pelvic reirradiation as a treatment option, with a significant proportion of papers reporting a median interval of over 12 months. A prolonged interval will enable normal tissue to repair and have a positive effect on the patient’s ability to tolerate retreatment. Compliance will be affected by the total number of fractions, frequency of dosing, total treatment volume, and concurrent use of capecitabine. Kishan et al. directly compared patients undergoing reirradiation to those receiving primary radiotherapy, concluding that there were no significant differences in toxicity profiles [15], an important factor to be discussed with patients when counselling them on treatment options.

All of the studies included in this review used radiotherapy as a neoadjuvant or palliative treatment. More robust data are needed to propose reirradiation as a first line alternative in surgically fit patients. This review does, however, highlight significant heterogeneity in the currently available data, with wide variation in reported outcomes, a lack of standardised definition of treatment success, high risk of bias, paucity of reporting of observed toxicity, and limited/inconsistent quality of life data. Where acceptable toxicity is reported, it is important to consider that this applies only to highly selective patients, and that long term toxicity may also be underestimated. In their study, Nordkamp et al. included 173 out of 377 patients undergoing pelvic reirradiation. The authors focused on surgical outcomes and overall-, re-recurrence, and distant metastases-free survival, and did not provide radiobiological data [17]. There is also wide variation in treatment approaches and dosing strategies, making direct comparison of data challenging. The definition of treatment response is also sparse, reflecting the lack of international consensus but also the varying indications, patient selection, and treatment intent of reirradiation. Furthermore, the currently published literature does not fully reflect the technical advances in radiotherapy planning and delivery that have been made. Whilst a variety of reirradiation modalities are utilised internationally, a consensus regarding data reporting, including cumulative irradiation doses and reported patient outcomes, would enable direct comparisons to be made.

This review has focused upon an area of clinical interest and much needed research in the complex and challenging management of locally recurrent rectal cancer. In the palliative setting, the reirradiation approach will likely be determined by patient fitness, presence of metastatic disease, prognosis, and the need to minimise early and late toxicity. In the neoadjuvant setting, however, a more standardised approach needs to be considered, particularly with respect to the impact on further surgery in terms of both morbidity and ability to achieve R0 resection.

This review demonstrates that although data supporting the utility of reirradiation as a first line treatment is encouraging both in terms of tumour response and toxicity, high-quality, prospective studies are needed to clarify these effects and better inform treatment guidelines to establish whether it is a viable alternative to curative surgery. No novel or practice-changing conclusions can currently be drawn from the data available. From the limited and heterogenous data available, it would appear that reirradiation is relatively safe and well tolerated in both neoadjuvant and palliative settings; however, more consistency of outcome reporting and a minimum dataset should be encouraged for future studies.

## Figures and Tables

**Table 1:** A summary of primary and reirradiation schedules for included studies.

Study	Cohort Size (and Time Interval of Recruitment)	Primary Radiotherapy Treatment/Dose	Time Interval Between Radiation (Months)	Reirradiation Radiotherapy Treatment Modality and Dose	Cumulative Radiotherapy Data	Location of Recurrent Disease	Retreatment Volume
**External Beam Radiotherapy (EBRT)**							
Susko et al. [13] USA	N = 33 (2000–2014)	Not reported	7 (<24) 26 (>24), median interval 39/12 (25—50/12)	N = 15 2D/3D CRT 30 Gy/16# N = 2 2D/3D CRT + IORT 30 Gy/16# N = 11 IMRT 30 Gy/16# N = 5 IORT	Not available Cumulative median radiation dose = 77.4 (65.4–86.4 Gy)	N = 10 (30%) pre-sacral region N = 11 (33%) anastomotic N = 1 (3%) sacral N = 11 (33%) pelvic wall, uterus or bladder	“GTV + margin” but no further details available
Ng et al. [14] Australia	N = 56 (1997–2008)	Median total dose/fractionation 50.4 Gy/28# (range, 21–64 Gy). Rectal Ca (n = 52): no data provided Prostate Ca (n = 3): 64 Gy/32# Endometrial Ca (n = 1): 45 Gy/25# with 20 Gy brachytherapy boost EQD2 = median 49.6 Gy (44.3–58.4)	30, range 8–176 months	N = 39 (70%) EBRT 39.6 Gy/22# N = EBRT + IORT 10 Gy/1# Overall: 3D conformal (of which, 11% IMRT)	Cumulative dose: median 87.3 Gy, range 44.4–108 Gy	N = 21 Presacral N = 9 Pelvic sidewall N=8 Rectum N = 8 anastomosis N =8 Bladder N = 7 prostate N = 6 Vagina N = 5 Perineal scar N = 4 Rectal stump	GTV + 1 cm margin = CTV. CTV + 1 cm = PTV
Kishan et al. [15] USA	N = 25 (2005–2011)	Rectal Ca: n = 9 (36%): EQD2 = 49.6 Gy (range 44.3–50 Gy) Prostate Ca: n = 1 (4%): EDQ2 58.4 Gy	29.3	50% (n = 5) = IMRT 50% = 3D CRT Retreatment EQD2 = 30 Gy of which, n = 4 (40%) also received IORT (electron median dose 12 Gy, range 8–13 Gy)	Median EQD2 79.6 Gy (tumour) and 77.8 Gy (late reacting normal tissue)	Not reported	Not reported
Cai et al. [16] China	N = 22 (2007–2012)	Technique not specified. Median 48.6 Gy, range 36–62 Gy	30 (range, 13–100 months)	N = 22 39 Gy/30# delivered twice daily/3 weeks IMRT	Cumulative EQD2 (assuming a/b = 3) = 82.14	N = 5 perirectal N = 7 presacral N = 7 internal iliac nodal N =5 perineum N = 1 external iliac nodal	GTV + 2–3 cm = PTV 77.3% patients had lesions > 3 cm
Nordkamp et al. [17] Netherlands/Sweden	N = 173 (2003–2017)	Not specified 30 Gy/15#	Not reported	Not reported	Not reported	Not reported	Not reported
Valentini et al. [18] Italy	N = 59 (1997–2001)	3D CRT (×2 lateral fields +/− posterior field) ≤55 Gy (range 30–74 Gy, median 50.4 Gy)	27 (range, 9–106 months)	3D CRT (×2 lateral fields +/− posterior) 30 Gy/25# to total volume + sequential 10.8 Gy/9# boost to GTV + 2cm) Total dose 40.8 Gy, delivered twice daily, over 3½ weeks.	Not reported	Not reported	GTV + 4 cm
**Stereotactic Ablative Radiotherapy (SABR)**							
Robinson et al. [19] UK	N = 17 (2015–2020)	Not specified N = 10 45 Gy/25# N = 4 50.4 Gy/28# N = 3 54 Gy/25#	48 (range 16–84)	N = 17 30 Gy/5#	Median retreatment EQD2 61 Gy	Site of largest GTV: N = 13 Lymph node N = 2 bone N = 2 Soft tissue	GTV + 5 mm Median: 8.7 cm^3^ (range 0.5–121.7)
Johnstone et al. [20] UK	N = 69 (2015–2020)	Not Specified N = 1 54 Gy/30# N = 8 50.4–53.2 Gy/28# N = 40 45–52.5 Gy/25# N = 1 25 Gy/5# + 9.5 Gy/5# boost N = 1 30.4 Gy/5# N = 7 25 Gy/5# N = 1 20 Gy/4#	Not reported	N = 69 30 Gy/5#	Median cumulative EQD2 (alpha/beta 3) = 97.2 (range 86.0–109.2)	Site of largest GTV: N = 59 lymph node N = 1 Perianastomotic N = 1 sacrum N = 1 Penis N = 7 R1	Median: 13.4 cm^3^ (0.6–121.7)
Dagoglu et al. [21] USA	N = 18 (2006–2012)	Not Specified median dose 50.4 Gy (range, 25–100.4 Gy)	Median 22 (range 15–336)	N = 18 24–40 Gy (median 25 Gy), 3–6# (median 5#)	Not reported	N = 12 (57.1%) pelvic wall N = 5 (23.8%) Presacral N = 2 (9.6%) Central N= 1 Presacral and pelvis side wall (4.8%) N = 1 Pubic area (4.8%)	Median tumour volume: 90.1 mm (36.8–1029.4)
**Heavy-charged particle therapy: Carbon ion radiotherapy (CIRT)**							
Shiba et al. [22] Japan	N = 7 (2011–2021)	C-ion beams 50 Gy (range 40–50)	Not documented	N = 5 73.6 Gy/16#/4 weeks N = 257.6 Gy/12#/3 weeks	Not reported	N = 4 Presacral N = 3 Side wall	GTV 15.6 cm^3^ (range 3.4–162.4)
Barcellini et al. [23] Italy	N = 14 (2014–2018)	IMRT Median 45 Gy (range, 45–76) n = 1 = additional brachytherapy boost (20 Gy)	65 (range, 14–139)	N = 14 Median total CIRT: 60 Gy/16# (range 35–76.8 Gy)	Not reported	N = 10 Presacral N = 1 Perineal N = 1 Perianal N = 2 Pre-coccygeal	GTV + 5–10 mm (CTV) + 13 mm (PTV) GTV median 154cm^3^ (range 7.2–360)
Chung et al. [24] Japan/Korea	N = 66 (2005–2019)	Not reported	Not reported	N = 35 CIRT 70.4 Gy/16# N = 9 3D CRT 50 Gy/25# N = 22 IMRT Median 50 Gy (range: 25–62.5 Gy, median number of fractions 25 (range, 3–33))	Not reported	N = 27 Presacral N = 39 Nodal	CRT: GTV+ 5 mm Median tumour size 2.5 cm^3^ (range 15–80) EBRT: GTV + 0.5–3 cm (PTV) Median tumour size 3.0 cm^3^ (range 10–70)
Cai et al. [25] China	N = 25 (2015–2019) N = 17 patients with history of previous radiotherapy to same site	Not specified 27–60 Gy (median 50 Gy)	Not reported	N = 17 CIRT 48–75.6 Gy (median 72 Gy)/16–21#	Not reported	N = 11 presacral N = 9 side wall N = 4 perineal N = 1 perianastomosis	GTV + 5–10 mm (CTV) + 5 mm (PTV) 84.5 mL (6.1–334.1)
Habermehl et al. [26] Germany	N = 19 (2010–2013)	N = 17 EBRT 50.4–60.4 Gy (median 50.4 Gy) of which, N = 1, additional SABR 19 Gy reirradiation and a further N = 1 additional IORT 15 Gy	47.4 (17–110)	N = 19 CIRT 36–51 Gy/12-18#	Not reported	Not reported	Median PTV 456 mL (range, 75–1597 mL)
**Interstitial Brachytherapy**							
Bishop et al. [27] USA	20 (2000–2012)	Median total EBRT BED 90 Gy (72–149), with a total median BED (including IORT) of 115 Gy (range 72–225)	26	N = 20 interstitial brachytherapy: 80 Gy to tumour +1 cm, 120 Gy to GTV	Not reported	N = 10 presacral N = 6 sciatic notch N = 2 sidewall N = 2 other	37 mm (18–93)

CIRT, carbon ion radiotherapy; CRT, chemoradiotherapy; CTV, clinical target volume; EBRT, external beam radiotherapy; GTV, gross tumour volume; Gy, Gray; IMRT, intensity modulated radiotherapy; IORT, intra-operative radiation therapy; PTV, planning target volume; SABR, stereotactic ablative radiotherapy.

**Table 2:** A summary of outcomes of interest from papers included in this review.

Study	Local Control Rate	Overall Survival (3 Years)	Acute Toxicity Grade 3+ (N=)	Late Toxicities Grade 3+ (N=)
**External Beam Radiotherapy (EBRT)**				
Susko et al. [13]	15.7% (2 years)	30%	6% (2) n = 1 rectovesicular fistula n = 1 ureteral obstruction	21% (7) n = unspecified, ureteral obstruction, small bowel obstruction, enterocutaneous fistula
Ng et al. [14]	47% (3 years)	20% (2 years)	12% (7) n = unspecified	Not reported
Kishan et al. [15]	73.3% (3 years)	36%	0% (0)	0%
Cai et al. [16]	67% (1 year) 10.7% (2 years)	18%	22% (5) n = 2 diarrhoea n = 1 cystitis n = 1 radiation dermatitis n = 1 intestinal obstruction	Not reported
Nordkamp et al. [17]	72.8% (1 year) 41.8% (2 years) 34% (3 years)	55.6%	Not reported	Not reported
Valentini et al. [18]	38.8% (5 years)	61% (5 years)	5.1% (3) n = 3 gastrointestinal	12% (7) n = 2 skin fibrosis n = 2 male impotence n = 1 urinary incontinence n = 1 small bowel obstruction n = 1 dysuria
**Stereotactic Ablative Radiotherapy (SABR)**				
Robinson et al. [19]	Not reported	30%	Not reported	Not reported
Johnstone et al. [20]	28% (2 years)	65%	Not reported	Not reported
Dagoglu et al. [21]	85.9% (3 years)	59.3%	17% (3) n = 1 small bowel perforation n = 1 neuropathy n = 1 ureteric fibrosis	Not reported
**Heavy-charged particle therapy: Carbon ion radiotherapy (CIRT)**				
Shiba et al. [22]	83.3% (2 years)	57%	0% (0)	29% (2) n = 1 GI tract n = 1 Urinary
Barcellini et al. [23]	Not reported	76% (2 years)	0% (0)	0% (0)
Chung et al. [24]	Not reported	86%	0% (0)	18% (12) CIRT n = 2 GI tract CRT n = 4 GI tract CRT n = 2 GU
Cai et al. [25]	90.4% (1 year) 71.8% (2 years)	82.9% (1 year) 65.1% (2 years)	0% (0)	12% (3) n = 1 GI tract n = 1 Neuropathy n = 1 pelvic infection
Habermehl et al. [26]	Not reported	Not reported	0% (0)	0% (0)
**Interstitial Brachytherapy**				
Bishop et al. [27]	80% (1 year) 60% (2 years)	95% (1 year) 75%	Not reported	4% (1) n = 1 Ureteral stricture

CIRT, carbon ion radiotherapy; EBRT, external beam radiotherapy; SABR, stereotactic ablative radiotherapy.

## Data Availability

No new data were created or analyzed in this study. Data sharing is not applicable to this article.

## Supplementary Materials

The following supporting information can be downloaded at: https://www.mdpi.com/article/10.3390/biomedicines14061194/s1, Table S1. Expanded search strategy. Table S2. PRISMA checklist with manuscript references.
